# Antibacterial and Cytotoxic Efficacy of Extracellular Silver Nanoparticles Biofabricated from Chromium Reducing Novel OS4 Strain of *Stenotrophomonas maltophilia*

**DOI:** 10.1371/journal.pone.0059140

**Published:** 2013-03-21

**Authors:** Mohammad Oves, Mohammad Saghir Khan, Almas Zaidi, Arham S. Ahmed, Faheem Ahmed, Ejaz Ahmad, Asif Sherwani, Mohammad Owais, Ameer Azam

**Affiliations:** 1 Department of Agricultural Microbiology, Faculty of Agricultural Sciences, Aligarh Muslim University, Aligarh, India; 2 Center of Excellence in Materials Science (Nanomaterials), Department of Applied Physics. Aligarh Muslim University, Aligarh, India; 3 School of Nano and Advanced Materials Engineering, Changwon National University, Changwon, Republic of Korea; 4 Interdisciplinary Biotechnology Unit, Aligarh Muslim University, Aligarh, India; 5 Center of Nanotechnology, King Abdulaziz University, Jeddah, Saudi Arabia; RMIT University, Australia

## Abstract

Biofabricated metal nanoparticles are generally biocompatible, inexpensive, and ecofriendly, therefore, are used preferably in industries, medical and material science research. Considering the importance of biofabricated materials, we isolated, characterized and identified a novel bacterial strain OS4 of *Stenotrophomonas maltophilia* (GenBank: JN247637.1). At neutral pH, this Gram negative bacterial strain significantly reduced hexavalent chromium, an important heavy metal contaminant found in the tannery effluents and minings. Subsequently, even at room temperature the supernatant of log phase grown culture of strain OS4 also reduced silver nitrate (AgNO_3_) to generate nanoparticles (AgNPs). These AgNPs were further characterized by UV–visible, Nanophox particle size analyzer, XRD, SEM and FTIR. As evident from the FTIR data, plausibly the protein components of supernatant caused the reduction of AgNO_3_. The cuboid and homogenous AgNPs showed a characteristic UV-visible peak at 428 nm with average size of ∼93 nm. The XRD spectra exhibited the characteristic Bragg peaks of 111, 200, 220 and 311 facets of the face centred cubic symmetry of nanoparticles suggesting that these nanoparticles were crystalline in nature. From the nanoparticle release kinetics data, the rapid release of AgNPs was correlated with the particle size and increasing surface area of the nanoparticles. A highly significant antimicrobial activity against medically important bacteria by the biofabricated AgNPs was also revealed as decline in growth of *Staphylococcus aureus* (91%), *Escherichia coli* (69%) and *Serratia marcescens* (66%) substantially. Additionally, different cytotoxic assays showed no toxicity of AgNPs to liver function, RBCs, splenocytes and HeLa cells, hence these particles were safe to use. Therefore, this novel bacterial strain OS4 is likely to provide broad spectrum benefits for curing chromium polluted sites, for biofabrication of AgNPs and ultimately in the nanoparticle based drug formulation for the treatment of infectious diseases.

## Introduction

Chromium (Cr) used in the leather industry to tan hides is not taken up completely by leather and hence, relatively large amounts of Cr escapes into the effluent. The south eastern part of the largest industrial city of India, Kanpur, near the south bank of river Ganga, has more than 350 tanneries. The lack of stringent laws and treatment facilities has led to tannery wastes/sludge being dumped on temporary sites leading to the contamination of soil and ground water with serious environmental impacts during leather processing [1]. The toxicological impact of Cr(VI) originates from the action of this form itself as an oxidizing agent, as well as from the formation of free radicals during the reduction of Cr(VI) to Cr(III) occurring inside the cell. Cr is a toxic element to higher vascular plants and is detrimental to its growth, development and reproduction [2]. Mine workers are habitually exposed to Cr(VI) contaminated dust and water and, therefore, suffer from gastrointestinal bleeding, tuberculosis, asthma, infertility and birth defects. In some cases, twenty times the international standard for Cr(VI) has been detected in drinking water. Due to certain difficulties associated with physicochemical methods adapted for detoxification/remediation of Cr contaminated sites, biological treatments in recent times have received greater attention being an economical and environment friendly as compared to conventional technologies [3]. The bioremediation strategy involves the conversion of Cr(VI) into less toxic and less mobile Cr(III), which consequently is immobilized in the soil matrix [4]. In this context, many microbes have been reported to reduce Cr(VI) under aerobic and anaerobic conditions [5]–[7].

Nanomaterials synthesized so far, on other hand, have shown some profound effect on human health, economic growth and the environment. The development of techniques for the controlled production of nanoparticles of well-defined size, shape and composition, to be used in biomedical sciences, optics and electronics, is however, still a major challenge [8]. Therefore, realizing the undeniable magnitude of this technology and its consequential impact on human health and the environment, various governments and private sectors at global level have significantly increased their fundings in vital sectors like medicine, manufacturing, energy and transportation. Following this, the material science research in recent times has achieved considerable importance due to the unique property of nanoparticle size and shape [9]. Also, the inherent characteristics of nanoparticles such as high surface to volume ratios and quantum confinement result in materials that are qualitatively different from their bulk counterparts [10]. Generally, nanoparticles are synthesized by different physico-chemical methods [11]–[13], which are however, expensive and environment disruptive. Therefore, interest is growing every day to develop inexpensive, clean, non-toxic and eco-friendly strategies for the synthesis of nanoparticles with broad spectrum activity. In this regard, the advent of fabrication of bio-based advanced nanomaterials has provided solutions to the problems. For example, fabricated materials are now being used as/in (i) catalysts in chemical reactions [14] (ii) antibacterial agents [15], [16] (iii) biosensors [17] (iv) cancer therapy [18] and (v) bio-control agents [19] etc. Recently, eco-friendly synthetic chemistry approaches have however, involved some biological systems such as yeast [20], fungi [21], bacteria [22] and plant extracts [23] for the synthesis of nanoparticles. Some of the most commonly used microorganisms for developing microbes based silver nanoparticles (AgNPs) include fungi like *Fusarium* spp. [24] and *Aspergillus* spp. [25] and bacteria such as *Enterobacteria* spp. [26], *Pseudomonas* spp. [27], *Bacillus* spp. [28], and *Geobacter* spp. [29]. Mechanistically, the ability of bacterial strain to reduce nitrate [30] has been exploited in the reduction of silver nitrate (AgNO_3_) in to elemental nanomaterial [31].

Considering the significance of bio-based fabricated nanomaterials, the present study was designed to find bacterial strain originating from the heavy metal contaminated sites and to characterize the strain through molecular and biochemical approaches. The bacterial strain was further tested for its chromium and nitrate reducing ability. The bacterial strain was also used to synthesize AgNPs at room temperature in the absence of any reducing agent. The resulting AgNPs were subsequently characterized using some of the standard analytical techniques like, UV–visible, Nanoparticle Size Analyzer, SEM, XRD and FTIR spectroscopy. In addition to the antibacterial activity on both Gram-positive and Gram-negative bacteria, the cytotoxicity of biofabricated AgNPs was tested on liver function, RBCs, splenocytes and HeLa cell lines.

## Materials and Methods

### Isolation and Bacterial Characterization

The soil samples were collected in sterile polythene bags (15–12 cm^2^) from the rhizosphere of sweet pea (*Pisum sativum*) fields located at the outskirts of Ghaziabad, Uttar Pradesh, India. Historically, the agronomic field was irrigated consistently by industrial sewage water of Hindon river. In order to isolate the bacterial strain, a serial dilution assay was carried out in normal saline solution and 10 µL of diluted suspension was spread plated on nutrient agar (NA) medium. Plates were incubated at 28±2°C for three days. A total of 20 bacterial strains were selected and characterized. Biochemical activities were tested through citrate utilization, indole production, methyl red test, nitrate reduction, Voges Proskauer, catalase test, oxidase carbohydrates (dextrose, mannitol and sucrose) utilization, starch hydrolysis, and gelatin liquefaction test [32].

### 16S rDNA Based Identification

Out of the total 20 bacterial strains, strain OS4 was identified by 16S rDNA gene sequence analysis. The partial sequencing of 16S rDNA of the strain OS4 was done commercially by Sequencing Service, Macrogen Inc., Seoul, South Korea using universal primers, 518F (5′CCAGCAGCCGCGGTAATACG3′) and 800R (5′TACCAGGGTATCTAATCC3′). Later, nucleotide sequence data were deposited in the Gen-Bank, NCBI sequence database. The online NCBI program nBLAST was employed to identify the related sequences with known taxonomic information already present at NCBI website (http://www.ncbi.nlm.nih.gov/BLAST) to accurately identify the bacterial strain OS4. Phylogenetic tree was constructed by the neighbour-joining method [33] of the MEGA 4.1 software programme [34].

### Optimization of Growth and Hexavalent Chromium Reduction Conditions

The effect of viable bacterial populations and pH on hexavalent chromium, Cr(VI), reduction was assessed using nutrient broth (NB) amended with 100 µg ml^−1^ of Cr^6+^. The sterilized medium was adjusted to pH 2 to 12 with 1 M HCL or 1 M NaOH. A-100 µl of exponentially grown culture of *S. maltophilia* OS4 was inoculated into NB medium containing upto100 µgml^−1^ of Cr(VI) and incubated at 35±2°C in an orbital shaking incubator at 120 rpm upto 48 h. For Cr^6+^ reduction, 1 ml culture from each flask was centrifuged (6000 rpm) for 10 min at 20°C, and Cr^6+^ in the supernatant was determined by the 1,5–diphenyl carbazide method [35], [36].

### Medium and Growth Conditions for Supernatant Preparation

The bacterial isolate OS4 was inoculated in sterile NB medium (pH 7.2). Bacteria were allowed to grow at 35±2°C for 24 h in a 500 ml Erlenmeyer flask with working volume of 300 ml with agitation at 120 rpm on orbital shaking incubator (Remi CIS 24BL, India). Culture medium was then centrifuged at 5000 rpm to obtain cell-free supernatant [37].

### Preparation and Characterization of AgNPs

**Figure 1:**
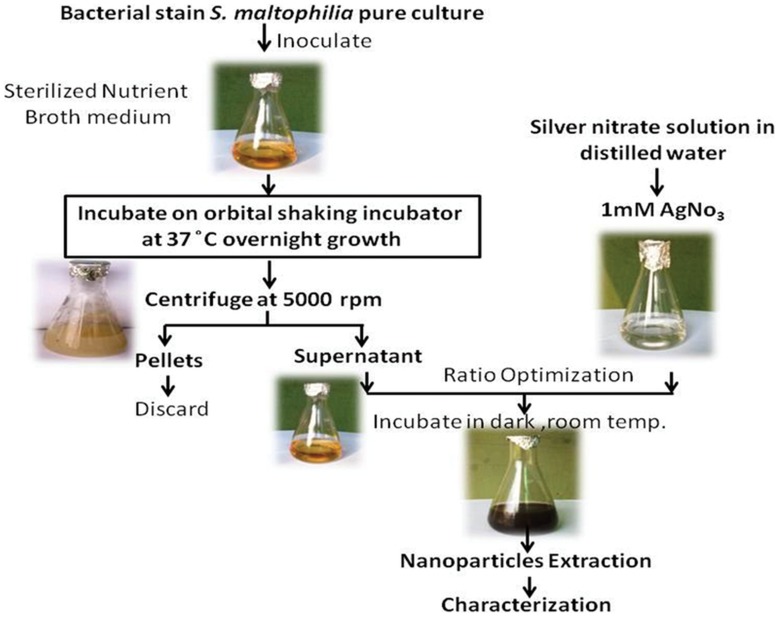
Outline of AgNPs biofabrication by supernatant of *S. maltophilia* OS4 strain. For more detail please see Materials and Methods.

To obtain AgNPs, 2 ml supernatant extracted from exponentially grown bacterial culture was added to 98 ml of 1 mM AgNO_3_ solution [38]. The reaction mixture was incubated in dark at room temperature. All steps in sequential manner are given in Figure 1.

### Purification of AgNPs

Bacterial supernatant was used for the synthesis of AgNPs. Bio-reduction of silver was monitored by the UV-vis absorption spectra as a function of time of the reaction mixture. The synthesized particles were washed six times by centrifugation and redispersed in double distilled water to remove the remaining unconverted silver ions. They were transferred into a dialysis bag with the cutoff of 12 kDa. AgNPs were resuspended in 1 ml of HEPES buffer (20 mM, pH 7.4) supplemented with sucrose to reach a density of 2.5 g/ml. The solution was placed at the bottom of a centrifuge tube (15 ml). A 12 ml of a linear gradient of sucrose (0.25–1.0 M) density was layered on the nanoparticle suspension and subjected to centrifugation (10,000 rpm at 4°C for 4 h) and were collected for further characterization. The concentration of nanoparticle in solution was determined by ICP-AES (Liberty RL) [39].

### UV- visible and Nanophox Spectra Analysis

The reduction of silver ions (Ag+) was carefully monitored by measuring the UV–visible spectrum of the reaction medium incubated overnight after diluting a small amount of aliquot prepared in double distilled water [40]. Since AgNPs are soluble in water, the change in colour was observed. A yellowish brown colour formation was noticed during the synthesis phase. The concentration of AgNPs produced was measured using a UV–vis spectrometer (Thermo Spectronic 20D+) between 250 and 600 nm wavelength, using 10-mm-optical-path-length quartz cuvettes. Further analyses of nanoparticles distribution and stability in solution were observed by the Nanophox particle size analyser [18].

### FTIR Spectra Analysis

RXI FTIR spectrometer with KBr beamsplitter was used to ascertain the involvement of biological moieties in particle synthesis. In order to remove any free biomass residue, the residual solution after the reaction was centrifuged at 8000 rpm for 30 min and the resulting pellets was mixed in 20 ml sterile double distilled water and cyclomixed for 10 min on vortex mixer. Thereafter, the centrifugation and re-dispersing process was repeated three times. The FTIR spectra of AgNO3, bacterial biomass and AgNPs were recorded as a KBr pellet at a resolution of 4 cm^−1^ in the wave number region of 400–4000 cm^−1^ [41].

### X-ray Diffraction and Field Emission Scanning Electron Microscopy Analysis

X-ray Diffraction analysis (XRD) was carried out using Rigaku Miniflex X-ray diffractometere with Cu-Kα radiations (λ = 0.15406 nm) in 2θ range from 20° to 80°. Furthermore, the morphology of the AgNPs was examined by field emission scanning electron microscopy (FESEM) on Hitachi SU6600. The sheet of the samples was prepared on a carbon coated copper grid by dropping a tiny amount of the sample and prior to measurements grids were allowed to dry at room temperature [42].

### Release Kinetics of AgNPs

AgNPs were placed in a dialysis bag with a cut off 12 kDa. The bag was suspended in a HEPES buffer solution (20 ml, pH 7.4). A continuous release of AgNPs was measured over a period of 48 h. The release rate and order of kinetics were calculated. The concentration of silver released was determined by atomic absorption spectrophotometer (GBC, Australia), Briefly, the solution of AgNPs was processed at 100°C in concentrated nitric acid in an oil bath for 2 h for complete solvation and then the silver concentration was measured by Air/Acetylene Flame via using silver flame, Fuel flow rate 0.9 to 1.2 L/min and atomize temperature 1100°C [43]. The percentage of silver released was calculated using equation:
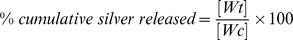
where Wc is the total silver content in the dialysis bag and Wt is the silver content in the buffer solution at a time.

### Animal Model for Efficacy and Toxicity Studies

For acute toxicity tests, female BALB/c mice weighing 18±2 g and 8–10 weeks old were obtained from the animal house facility of Interdisciplinary Biotechnology Unit, A.M.U., Aligarh. The animals were fed a standard pellet diet and had access to water *ad libitum.* The techniques used for bleeding and injection were followed strictly in accordance with mandates approved by the Government of India animal ethics committee for the control and supervision of experiments on animals.

### Cell Preparation

The spleen cells was isolated from mice and teased apart in RPMI 1640 medium. After centrifugation, the single cell suspension was treated with ACK-lysing buffer (8.29 g/L NH4Cl, 1 g/L KHCO2, 37.2 mg/L EDTA/2Na) to lyses red blood cells. After centrifugation, cells were maintained in RPMI 1640 medium supplemented with antibiotic and antimycotic solution (Sigma) and 10% heat inactivated fetal calf serum (FCS; sigma). Cells were cultured in a 96-well flat-bottomed plates at well in 0.1 ml of culture medium for cell proliferation assay, and stimulated with AgNPs (0–500 µgml^−1^). Splenocytes were cultured for 24 h at 37°C in a humidified atmosphere containing 5% CO_2_ and 95% air. Here, the used splenocytes were pre-activated by using 5 µg/ml of ConA for 48 h of incubation.

### Determination of AgNP Uptake by HeLa Cells

The HeLa cell line was maintained in RPMI 1640 culture medium supplemented with 10% heat-inactivated fetal calf serum. The cells were plated at a density of 10^4^ cells on glass coverslips, and cultured for 24 h at 37°C. The cells were subsequently exposed to AgNPs. The plates were incubated for 1 h. Cells were fixed by 2% paraformaldehyde for 2 h followed by washing with HBSS. The fixed cells were observed under microscope.

### MTT Assay on Splenocytes

The cytotoxicity of the AgNPs on murine splenocytes was determined by a previously reported method standardized in our laboratory [18]. After stimulation with AgNPs (0–500 µgml^−1^) for 24 h at 37°C, the splenocytes were centrifuged and washed twice with fresh RPMI 1640 medium and grown in 0.5 mgml^−1^ MTT (dissolved in PBS and filtered through a 0.2 mm membrane) at 37°C. After 4 h, the intracellular formazan crystals were dissolved in dimethylsulphoxide, and the absorption values were measured at 570 nm. The absorption values were expressed as the cell proliferation rate (%), according to the control group as 100%.

### MTT Assay on HeLa Cell Lines

The HeLa cell line was maintained in RPMI 1640 culture medium supplemented with 10% heat-inactivated fetal calf serum. The cells were plated at a density of 5×10^4^ cells per well in a U-bottom 96-well plate, and cultured for 24 h at 37°C. The cells were subsequently exposed to various concentrations of AgNPs (0–500 µgml^−1^). The plates were incubated for 48 h, and cell proliferation was measured by adding 20 µL of MTT (thiazolyl blue tetrazolium bromide) dye (5 mg/ml in phosphate-buffered saline) per well. The plates were incubated for a further 4 h at 37°C in a humidified chamber containing 5% CO_2_. Formazan crystals formed due to reduction of dye by viable cells in each well were dissolved in 150 µL dimethyl sulfoxide, and absorbance was read at 570 nm. The absorption values were expressed as the cell proliferation rate (%), according to the control group as 100%.

### Acute Toxicity Test for AgNPs

Hepatic toxicity was monitored by applying single dose regimen of 100 µg/ml to determine the biochemical profiles of serum aspartate aminotransferase (AST) and alanine aminotransferase (ALT) by respective detection kits (COGENT, Span Diagnostics Ltd., India). Blood was collected by retro orbital puncture from mice of two groups including (i) untreated and (ii) treated after 24 h. The blood was allowed to clot at room temperature and serum was separated. Aspartate aminotransferase and alanine aminotransferase was determined in serum as per respective guidelines provided by the manufacturer.

### RBC Lysis Test for AgNPs

*In vitro* erythrocyte lysis test was carried out as a preliminary toxicity test, which is assessed by measuring the hemoglobin released as a result of membrane leakage or disruption caused by exposure to low doses of the nanoparticles. Briefly, fresh blood obtained from a healthy rabbit was collected in anticoagulant solution (ethylene diamine tetra acetic acid) and centrifuged at 1000×g for 10 min at 4°C. Both Buffy coat and plasma were discarded. Washed erythrocytes were diluted with isotonic buffer (20 mM PBS) to prepare 50% hematocrit. Extent of hemolysis was studied by incubating the RBC suspension with various concentrations ranging from 100 to 300 µg/ml of AgNPs at 37°C for 1 h. The incubated solutions were centrifuged at 1500×g after 1 h and supernatant was collected and analyzed by ultraviolet-visible spectroscopy (λ_max_ = 576 nm) for released hemoglobin. The percentage hemolysis was determined by the following equation:
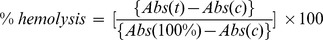
where Abs_t_ is the absorbance of the supernatant from samples incubated with the particles, Abs_c_ is the absorbance of the supernatant from controls (normal saline), and Abs100% is the absorbance of the supernatant of controls incubated in the presence of 1% Triton® X-100, which causes complete lysis of RBCs (total lysis).

### Antimicrobial Assay

The biofabricated AgNPs were tested for bactericidal activity by agar well-diffusion method against both Gram positive *Staphylococcus aureus* and Gram negative *Escherichia coli and* Serratia marcescens. The pure culture of each bacterium was sub-cultured in NB medium. Each bacterial strain was spread uniformly onto the individual plates by using sterile glass rod spreader. Wells of 8 mm diameter were punched into NA plates using gel puncture. By using a micropipette, nanoparticle (12.5, 25 and 50 µg) suspensions were poured into each well on all plates. Plates were then incubated at 35±2°C for 48 h and the level of zone of inhibition of bacterial growth was measured [44].

## Results and Discussion

### Characterization of Bacterial Strain

**Figure 2:**
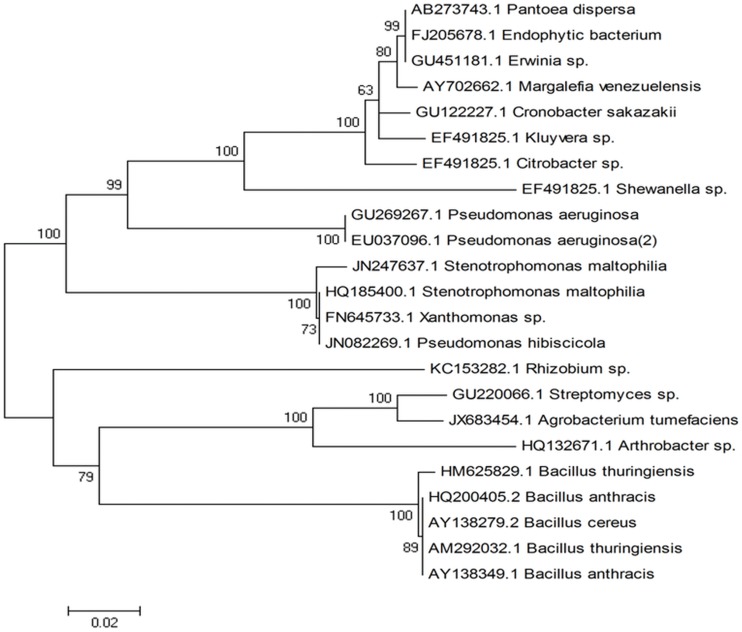
Phylogenetic tree constructed from the 16S rDNA sequence of *S. maltophilia* OS4 strain (GenBank: JN247637.1) with other related microorganisms by using Mega4 software.

In the present study, Cr(VI) resistant bacterium was isolated from industrial effluents contaminated soil. Out of the 20 bacterial isolates, OS4 strain was selected especially due to its ability to tolerate high level of most toxic form of chromium and was characterized morphologically and biochemically (Table 1). Strain OS4 grew well on NA plates amended with 1200 µg K_2_Cr_2_O_7_/ml. The chromium resistant bacterial strain was found to be Gram-negative, rod shaped and produced green pigments on NA plates. The freshly grown cultures showed a positive reaction for citrate utilization, nitrate reduction, catalase, and could hydrolyze starch and gelatin, but were negative for other biochemical tests (Table 1). On the basis of the characteristics observed for strain OS4 and compared with those listed in Bergey`s Manual of Determinative Bacteriology [32], strain OS4 was presumptively identified as *Stenotrophomonas* spp. In order to further validate strain OS4 and to identify the bacterial species, it was subjected to 16S rDNA sequence analysis. The sequence of 16S rDNA of strain OS4 was submitted to Gen-Bank (entry code: JN247637). The information obtained by the BLAST program indicated a close genetic relatedness of strain OS4 with the rDNA sequence of *S. maltophilia* (16S: 99% similarity with the reference sequence HQ185400.1) in NCBI database. Such a higher identical value confirmed the strain OS4 to be *Stenotrophomonas maltophilia.* A phylogenetic tree constructed by MEGA4 software based on 16S rDNA partial sequence is presented in Figure 2. Microorganisms in general have been found to survive in metal contaminated environment [45] and this property of metal tolerance by microbes have been/being exploited well in the bioremediation strategies to clean up contaminated sites [46]. Some of the strategies adopted by bacterial populations to protect themselves from the nuisance of metal toxicity include- (i) restriction of metal entry in to the cell either by reduced uptake/active efflux or by the formation of complexes outside the cell (ii) avoidance and sequestrations and (iii) enzymatic reduction of free ions in the cytosol. Despite these mechanisms, the information on the impact of Cr(VI) on bacterial populations inhabiting contaminated environment is contradictory. As an example, the Gram positive *Bacillus* strains tolerated chromium up to the concentration of 500 (PSB1), 400 (PSB7), and 550 µg ml^–1^ (PSB10), respectively, when grown on chromium amended NA plates [45] while other Gram-positive bacterium *Bacillus sphaericus* isolated from serpentine soil could tolerate 800 µg ml^−1^ Cr (VI) [47]. The differential response of bacterial cells even within the same group is likely due to the variation in the compositions of medium used or variable growth conditions [48]. However, whatever have been the reasons; the bacterial strain isolated in this study exhibited a high level of tolerance to Cr(VI) which could be an advantage while using this strain under chromium stressed soils.

**Table 1:** Morphological and biochemical characteristics of Cr(VI) resistant *Stenotrophomonas maltophilia* OS4 strain.

Features	Specifications	Outcomes
Appearance	Shape	Short Rod
	Pigments	+
	Gram reaction	–
Biochemical reactions	Citrate utilization	+
	Indole	–
	Methyl red	–
	Nitrate reduction	+
	Catalase	+
	Oxidase	–
	Voges Proskauer	–
Carbohydrate utilization	Glucose	+
	Mannitol	–
	Sucrose	+
Hydrolysis	Starch	+
	Gelatin	+
Tolerance	Cr(VI)	1,200 µg/ml

### Chromium Reduction Assay

**Figure 3:**
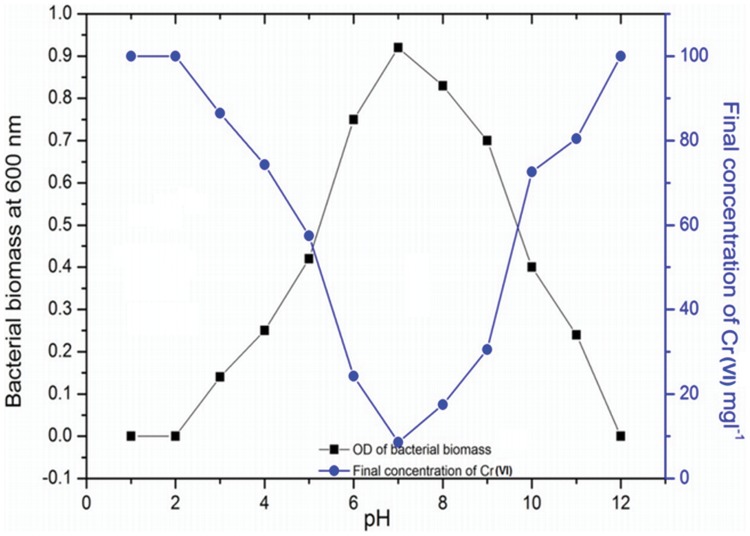
Determination of bacterial growth by taking the absorbance at 600 nm and observation of chromium reduction by *S. maltophilia* OS4 in response to various pH at 35±2°C temperature after 48 h incubation. For more detail, please see the Materials and Methods.

The effect of pH on chromium reduction by exponentially grown bacterial strain OS4 was variable (Figure 3).The bacterial strain grew well at pH 7 and could remove hexvalent chromium maximally by 91% after 48 h growth. However, with increase or decrease in pH, there was a corresponding decrease in bacterial growth which subsequently affected the reduction of Cr(VI) very negatively (Figure 3). For example, a maximum decrease (100%) in chromium reduction by strain OS4 was observed at pH 2 compared to those recorded at pH 7. In a similar study, Wani et al. [45] have also observed a variable effect of pH on chromium reduction by *Bacillus* spp. grown in NB treated differently with varying concentrations of Cr(VI). The chromium reducing ability of strain OS4 thus suggests that this strain might have enzyme chromium reductase which possibly led to the reduction of chromium, as also reported by Farrel and Ronallo [49].

### Characterization of Biofabricated AgNPs

**Figure 4:**
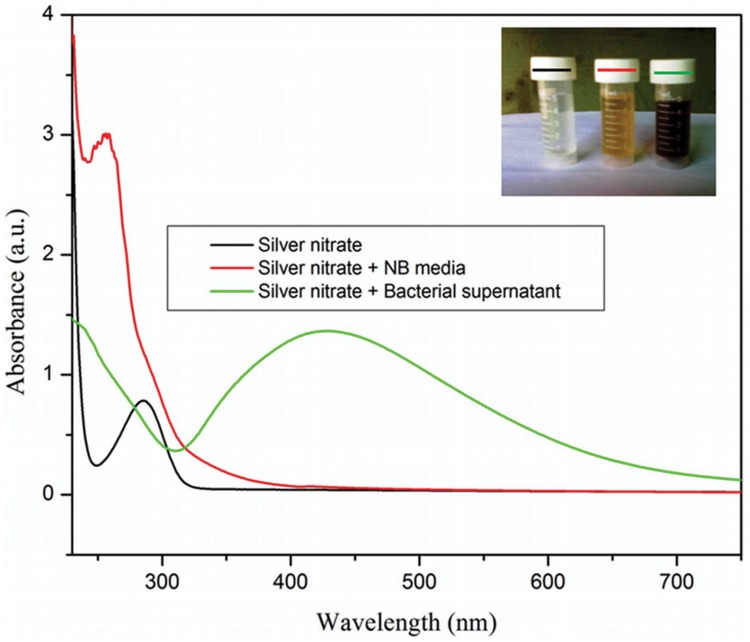
UV-visible spectrum of 1 mM aqueous solution of AgNO_3_ (black), AgNO_3_ in NB media (red) and AgNO_3_ in bacterial culture supernatant (green). The curves are recorded after a period of 30 min incubation. (Inset) 1 mM aqueous solution of AgNO_3_ (black lined cap), AgNO_3_ in NB media (red lined cap) and 30 min incubated reaction mixture of AgNO_3_ and supernatant of log phase of OS4 strain (green lined cap). For more detail, please see the Materials and Methods.

**Figure 5:**
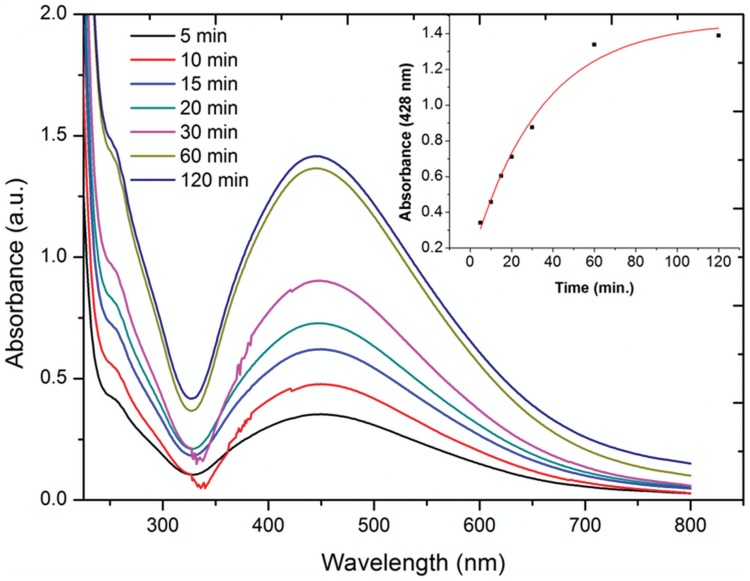
Absorption spectra profile of time dependent AgNP formation obtained from wavelength in nm plotted against absorbance. Kinetics of AgNP formation by plotting absorbance at 428 nm *vs* time in min is shown in inset. For more detail, please see the Materials and Methods.

**Figure 6:**
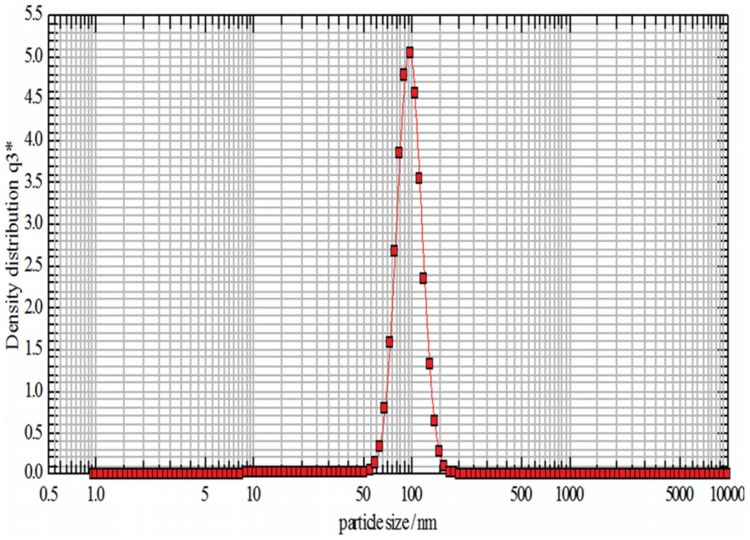
Size distribution curve of nanoparticles plotted against particle size in nm *vs* density distribution in reaction mixture obtained by Nanophox particle size analyser. For more detail, please see the Materials and Methods.

**Figure 7:**
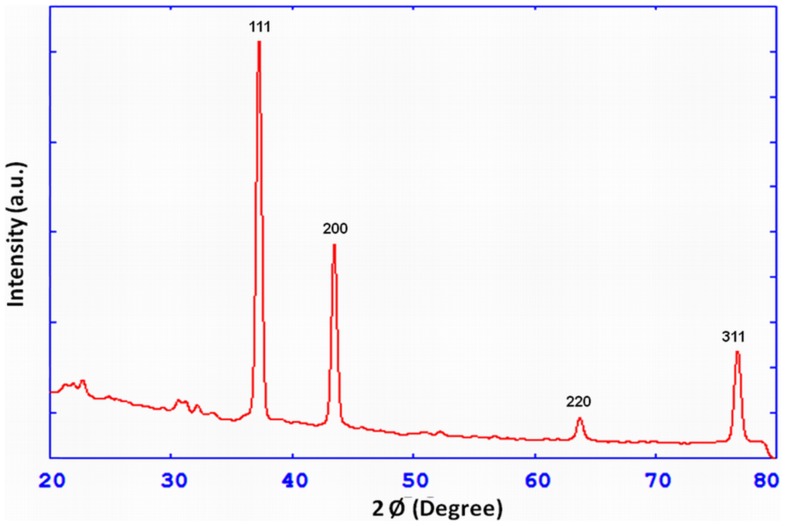
X-ray diffractogram of biosynthesized AgNPs plotted against 2θ in degree vs intensity. For more detail, please see the Materials and Methods.

**Figure 8:**
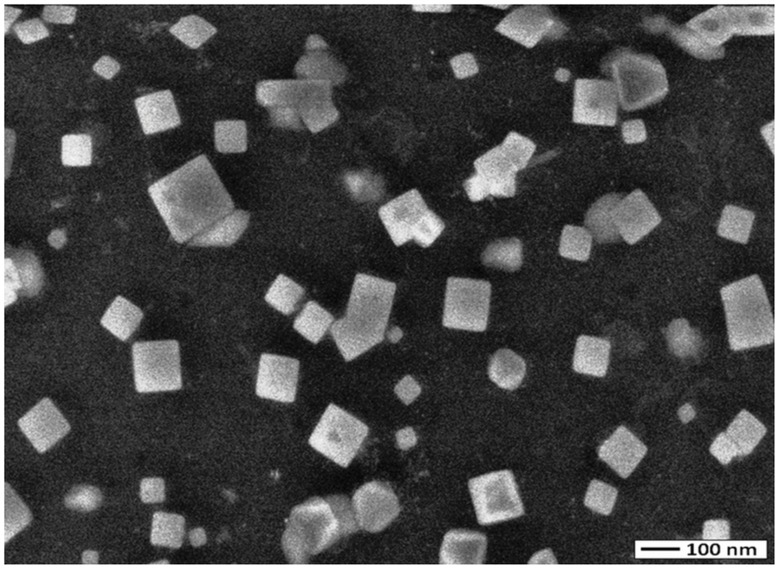
FESEM image of biogenic nanoparticles by applying the aqueous samples on a carbon coated grid and dried at room temperature. For more detail, please see the Materials and Methods.

**Figure 9:**
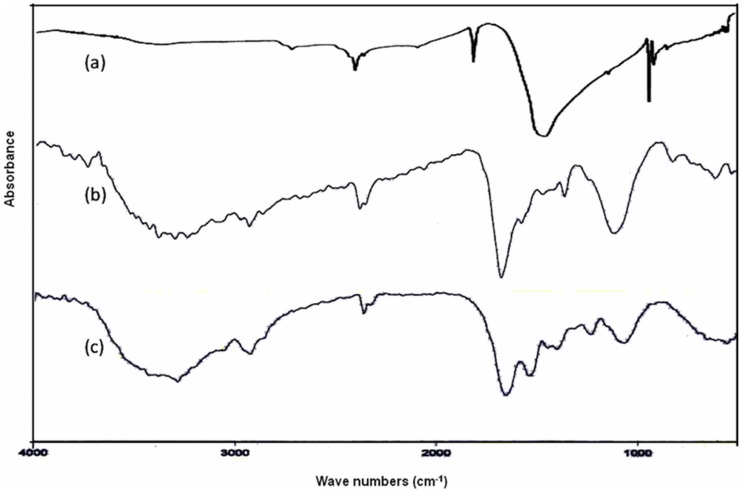
FTIR spectra of (a) AgNO_3_, (b) bacterial biomass and (c) AgNPs recorded as a KBr pellet at a resolution of 4 cm^−1^. For more detail, please see the Materials and Methods.

The colour of reaction mixture changed from colourless to yellowish brown in 30 min when the supernatant prepared from strain OS4 was added to solution of AgNO_3_. The intensity of colour further increased with increasing incubation periods. Formation of AgNPs using AgNO_3_ solution was confirmed by UV–visible spectral analysis. In the UV–visible absorption spectrum, there was lack of any characteristic peak of nanoparticle for AgNO_3_ in aqueous solution and AgNO_3_ in NB media while a strong and broad peak located at about 428 nm was observed for the synthesized AgNPs (Figure 4). Such type of UV-visible peak is in accordance to other previously reported various metal nanoparticles of 6 to 100 nm size [50], [51]. To check the effect of time on the synthesis of AgNPs we recorded time dependent UV-visible absorption spectra and plotted the absorbance intensity at 428 nm against time (min) to characterize the kinetics of AgNP formation (Figure 5). We found that within 2 h saturation was achieved. Furthermore, to check the size and stability of AgNPs in suspension, the particle size analyzer was also used. The size of colloidal AgNPs from particle size distribution curve was found to be 93 nm (Figure 6) which were in good agreement with the data observed under XRD, UV-visible spectroscopy and FESEM studies. The XRD analysis confirms the formation of single phase cubic AgNPs (Figure 7). All the peaks matched well with the standard JCPDS card No. 04-0783 of cubic AgNPs (Figure 8). Average particle size calculated from XRD data was found to be ∼93 nm which correlated well with the results obtained by particle size analyzer. Furthermore, the FESEM image of the synthesized AgNPs (Figure 9), validates the formation of cubic nanoparticles capped with its bio-moieties. This indicates the reduction of Ag^+^ to elemental silver (Ag). In other words, the UV-vis spectra show SPR for AgNPs at 428 nm and the cubical images observed in this study for NPs through SEM was in agreement to those reported earlier [51], [52]. Moreover, the synthesized nanoparticles were stable in solution over a time period of three months time at room temperature.

Biofabrication of AgNPs by the bacterial supernatant depends on the biological functional groups present on the bacterial surface. Hence, in order to understand better the kind of information about the functional groups involved in the capping or reduction process, FTIR analysis of the AgNPs in the range of 1000–4000 cm^−1^ was performed (Figure 9). The bands characterization including hydroxyl and amine group peaks were assigned at 3376 cm^−1^, alkyl and CHO had a broad band ranging between 2967–2851 cm^−1^, C = O of amide groups at 1644 cm^−1^, COO^−^ of the carboxylate groups appeared at 1584 and 1544 cm^−1^, the band located at 1238, 1398 and 1740 cm^−1^ represented COO^−^ anions where as those located at 726 cm^−1^ was assigned SO_3_^−^ groups. The IR spectra revealed the role of proteins and enzymes involved in capping and reduction of Ag^+^ in the formation of nanoparticles similar to the earlier report of Kumar and Mamidyala [53]. The band at 1650 cm^−1^ arises due to carbonyl stretch and -NH stretch vibrations in the amide linkages, clearly indicating the presence of protein/peptide on the surface that appears to be acting as a capping/stabilizing agent [54], [55].

As AgNPs itself have unique antibacterial activities due to generation of free radicals in bacterial membrane and cause toxicity in bacterial cell, they were not used as a drug loading like liposome particles or other peptide particles.

To check the short- and long-term storage stability of AgNPs, the biosynthesized AgNPs were stored in the dark at 4°C and 25°C temperatures. The UV-vis spectra and particle size were recorded continuously for 2 h (Figure 4) and after three months. It was found that there was no change in the particle size distribution and absorbance spectra with time suggesting that the size of AgNPs were stable with the course of time.

### Kinetics of AgNP Release

**Figure 10:**
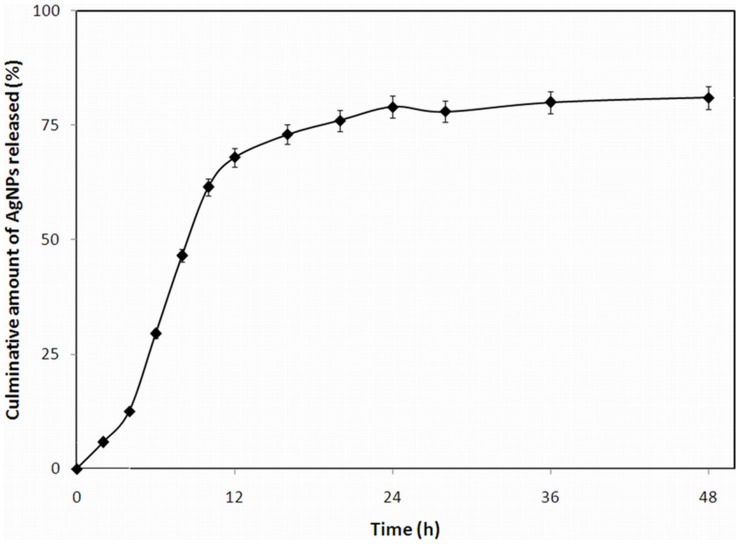
Curve for release kinetics of AgNPs plotted against time in h *vs* amount of nanoparticle in %. For more detail, please see the Materials and Methods.

The release kinetics of biosynthesized AgNps when placed in a dialysis bag and released into the medium through dialysis membrane is shown in the Figure 10 [56]. The measurement of sample was conducted using atomic absorption due to the low concentration of silver released in the early stages of the experiments. The method was very sensitive to recognizing the low concentration of the silver released especially at initial stage of the experiment. Continuous release of silver was observed over the period of the study (48 h). The rate of dissolution and the concentration of silver was obeying the zero order kinetics with r^2^>0.96. In accordance to the previous reports as observed with other drugs [57], the AgNPs that are released in to the medium are expressed as cumulative drug released % versus time (h). Throughout the course of the experiment, ∼81±3.7% of the AgNPs was released. This rapid release was correlated with the particle size and increasing surface area of the AgNPs. Therefore, the small sized nanoparticles showed a more rapid release kinetics than the bigger sized nanoparticles probably due to higher diffusability of the small particles.

### Cellular uptake by HeLa Cells and Cytotoxic Potential of AgNPs

**Figure 11:**
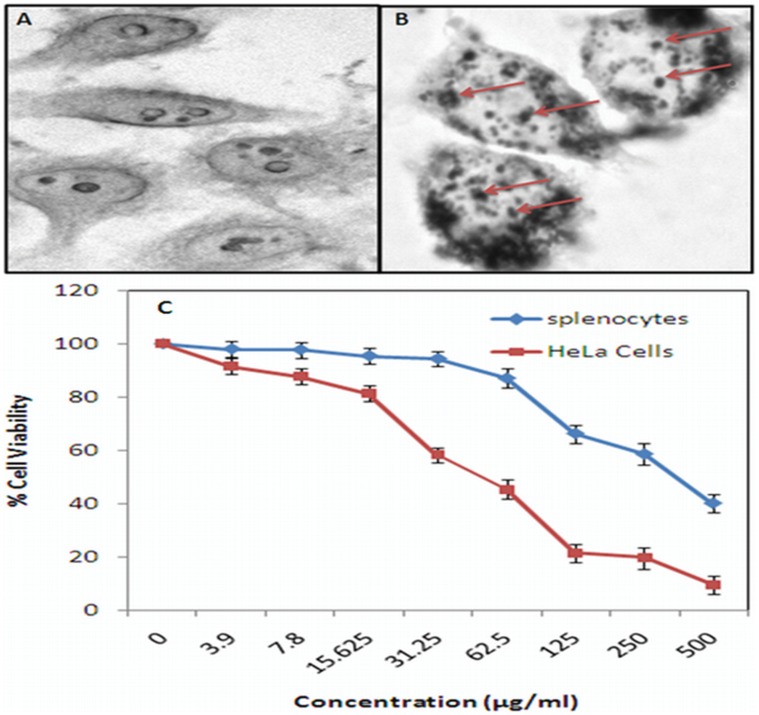
Bright field micrographs, captured after 2 h of incubation, showing endocytosis mediated-uptake of AgNPs by HeLa cells (A) without and (B) with AgNPs (500 µµg/ml). (C) MTT assay for cytotoxic effect of AgNPs against splenocytes and HeLa cell lines. Cells were dispensed at the density of 5×10^4^ cells per well in U-bottom 96 well plates in triplicate, and treated with increasing concentration of AgNPs (0–500 µg/ml). After 48 h of incubation, 20 µl of MTT reagent was added and plate was further incubated for 4 h followed by addition of 150 ml of dimethyloxide and the plate reading was carried out at 570 nm.

The uptake of in-house prepared AgNPs was assessed for HeLa cells. The incubation of HeLa cells with AgNPs resulted in nanoparticle uptake by endocytosis which mainly appeared in the aggregated form within the cells (Figure 11 A and B).

The cytotoxic potential of the synthesized AgNPs against HeLa cells and swiss mice splenocytes was assessed by determining the number of viable cells surviving after incubation with the AgNPs for the stipulated time period using the MTT method [18]. In other words, MTT assay was used to determine the differential cytotoxicity of AgNPs against both cell line and splenocytes. The cytotoxicity assay suggests a variable cytotoxicity of AgNPs for HeLa cells and splenocytes. AgNPs were found to be significantly more toxic to HeLa cells as compared to mouse splenocytes which can be attributed to the intrinsic anticancer property of AgNPs (Figure 11C).

### In vitro and in vivo Toxicity of AgNPs

**Figure 12:**
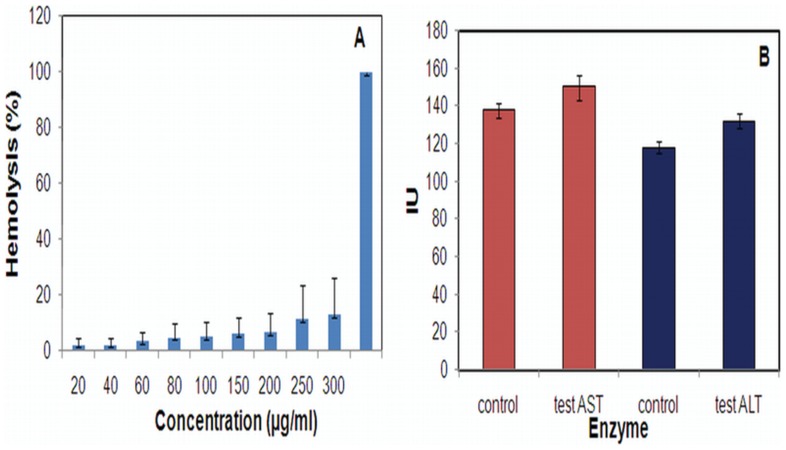
Determination of intrinsic toxicity of AgNPs. (A) *in vitro* induction of RBC hemolysis in presence of AgNPs (0–300 µg/ml) and control 1% Triton®X-100. (B) *in vivo* toxicity of AgNPs analyzed by liver function test (LFT) for marker enzymes AST and ALT in test (animals treated with AgNPs) and control (untreated) animals. For more detail, please see Materials and Methods.

We also evaluated the intrinsic toxicity of the generated AgNPs *in vitro* as well as *in vivo*. The test particles induced significantly less hemolysis as compared to control (1% Triton®X-100) which was ascertained when a 300 µg/ml solution of nanoparticles rendered merely 13% RBC hemolysis (Figure 12A). In another set of experiments, AgNPs administered animals were analyzed for liver function test parameters to evaluate *in vivo* toxicity. As shown in Figure 12B, animals which were given AgNPs had marginally higher levels of marker enzymes, AST and ALT, than untreated control animals. The results obtained here thus consolidated the facts that the in-house prepared AgNPs did not show any *in vitro* toxicity and even the *in vivo* toxicity was highly insignificant and hence such particles are safe to use in drug formulation.

### Antimicrobial Activity

**Figure 13:**
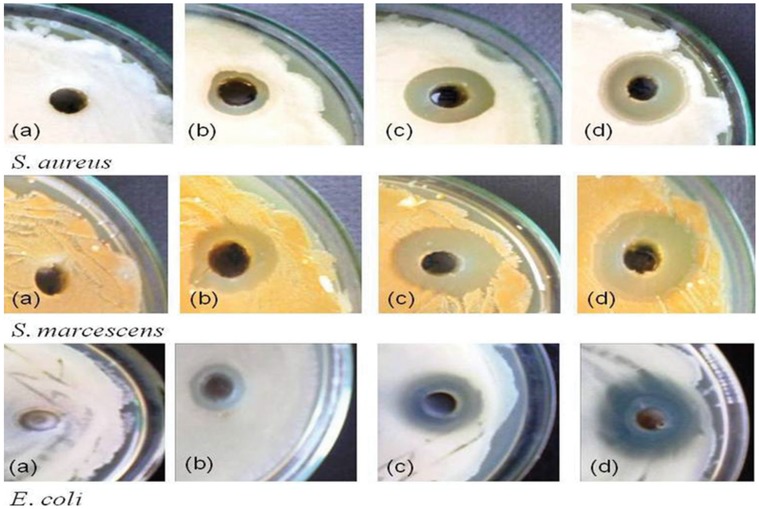
Antibacterial activities of biosynthesized AgNPs against Gram positive *S. aureus* and Gram negative *S. mercescens* and *E. coli* as (a) control; (b) 12.5 µµg AgNPs; (c) 25 µµg AgNPs and (d) 50 µµg AgNPs. For more detail, please see the Materials and Methods.

**Figure 14:**
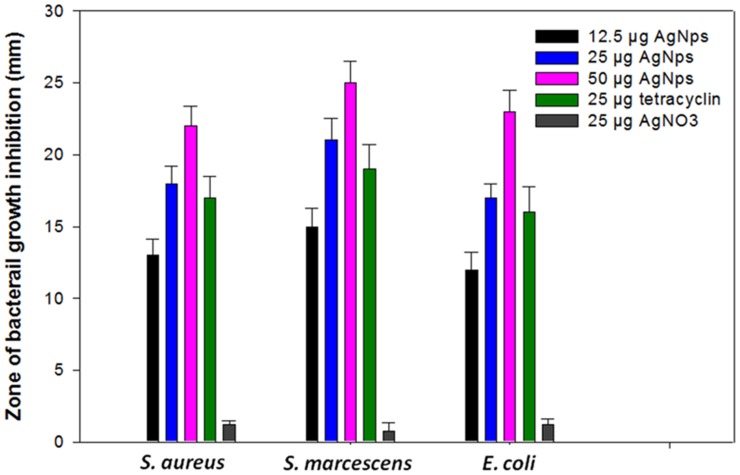
Effect of different concentrations of AgNPs (0–50 µµg), tetracycline (25 µµg) and AgNO_3_ powder (25 µµg) on bacterial growth. For more detail, please see the Materials and Methods.

After characterization, the newly synthesized AgNPs were tested for their antibacterial activity. Interestingly, different concentrations of the biofabricated AgNPs exhibited significant antibacterial activity against both Gram-negative (*S. marcescens* and *E. coli*) and Gram-positive (*S. aureus)* bacteria grown in NA medium. The antibacterial activity expressed in terms of zone of inhibition was quite visible on NA plates (Figure 13). Generally, the antibacterial activity of AgNPs increased considerably with corresponding increase in concentrations ranging from 12.5 µg to 50 µg per well. At these concentrations, the growth of bacterial strains namely *E. coli*, *S. aureus* and *S. marcescens* was declined significantly by 91, 69 and 66%, respectively (Figure 14). Upon loading of 50 µg AgNPs into 8 mm agar well, a maximum zone of inhibition was recorded for *S. marcescens* (25 mm), *E. coli* (23 mm) and *S. aureus* (22 mm). The bactericidal property of the nanoparticles suggested that these particles were more diffusible in the growth medium which in turn allowed greater interaction between bacterial cells and each nanoparticle. Similar bactericidal impact of some other Ag^+^ on microbial communities is known. For example, in many cases it has been proposed that ionic silver strongly interacts with thiol groups of vital enzymes and inactivates them. Also, there are evidences suggesting that DNA replication is halted when bacterial cells are exposed to Ag^+^ [58]. Even-though, we could not pinpoint the exact site where nanoparticles could affect the bacterial cells but it was very clear that Gram negative bacterial strains were most susceptible to nanoparticles [59].

### Conclusion

Due to chrome leather tanning processes, large quantities of chromium compounds are discharged through liquid, solid, and gaseous wastes into the environment and can have significant adverse biological and ecological effects. Conversion of Cr(VI) into less toxic Cr(III) is the main bioremediation strategy to overcome the chromium pollution. In this study, isolated and well characterized bacterial culture *S. maltophilia* exhibited chromium reducing ability that could help in cleanup of chromium contaminated environment. Furthermore, the culture supernatant of *S. maltophilia* strain OS4 was used for synthesis of AgNPs at room temperature using AgNO_3_. Since chromium reduction is the specific property of this strain, it is proposed that the reduction of Ag^+^ might be due to the protein component contributed by the enzyme reductase. It was confirmed that the AgNPs formed were cuboidal in shape, crystalline in nature with anionic particle surface. The AgNPs generated here also showed a promising antimicrobial activity against both Gram-positive and Gram-negative bacteria. On the contrary, AgNPs were found to be non-toxic in various cytotoxic assays. Thus, the bacterial strain OS4 used in this study is likely to provide broad spectrum benefits such as: (i) it may be helpful in curing chromium polluted sites; (ii) it could be used to generate biofabricated AgNPs and (iii) it could be effective in the execution of infectious diseases.
